# Advances in Translational Nanotechnology: Challenges and Opportunities

**DOI:** 10.3390/app10144881

**Published:** 2020

**Authors:** Shyam S. Mohapatra, Robert D. Frisina, Subhra Mohapatra, Kevin B. Sneed, Eleni Markoutsa, Tao Wang, Rinku Dutta, Ratka Damnjanovic, Manh-Huong Phan, Daniel J. Denmark, Manas R. Biswal, Andrew R. McGill, Ryan Green, Mark Howell, Payal Ghosh, Alejandro Gonzalez, Nadia Tasnim Ahmed, Brittney Borresen, Mitchell Farmer, Melissa Gaeta, Krishna Sharma, Christen Bouchard, Danielle Gamboni, Jamie Martin, Bianca Tolve, Mandip Singh, Jack W. Judy, Chenzhong Li, Swadeshmukul Santra, Sylvia Daunert, Elnaz Zeynaloo, Ryan M. Gelfand, Steven Lenhert, Eric S. McLamore, Dong Xiang, Victoria Morgan, Lisa E. Friedersdorf, Ratnesh Lal, Thomas J. Webster, David P. Hoogerheide, Thanh Duc Nguyen, Martin J. D’Souza, Mustafa Çulha, Pierre P. D. Kondiah, Donald K. Martin

**Affiliations:** 1 Taneja College of Pharmacy Graduate Programs, University of South Florida, Tampa, FL 33612, USA;; 2 Departments of Molecular Medicine and Internal Medicine, Morsani College of Medicine, University of South Florida, Tampa, FL 33612, USA;; 3 Department of Chemical and Biomedical Engineering and Global Center for Hearing and Speech Research, University of South Florida, Tampa, FL 33620, USA;; 4 Department of Physics, University of South Florida, Tampa, FL 33620, USA;; 5 James A. Haley Veterans Hospital, Tampa, FL 33612, USA; 6 College of Pharmacy and Pharmaceutical Sciences, Florida A&M University, Tallahassee, FL 32307, USA;; 7 University of Florida Department of Electrical and Computer Engineering and Nanoscience Institute for Medical and Engineering Technology, Gainesville, FL 32611, USA;; 8 Department of Biomedical Engineering, Florida International University, Miami, FL 33174, USA;; 9 NanoScience Technology Center, University of Central Florida, Burnett School of Biomedical Sciences, Department of Chemistry and Department of Materials Science and Engineering, Orlando, FL 32826, USA;; 10 Department of Biochemistry and Molecular Biology, University of Miami, Miller School of Medicine, and Department of Chemistry, Miami, FL 33124, USA;; 11 School of Science and Engineering, Tulane University, New Orleans, LA 70118, USA;; 12 Department of Biological Science, Florida State University, Tallahassee, FL 32306, USA;; 13 Agricultural and Biological Engineering, Institute of Food and Agricultural Sciences, University of Florida, Gainesville, FL 32603, USA;; 14 National Nanotechnology Coordination Office, Alexandria, VA 22314, USA;; 15 Center for Excellence in Nanomedicine and Engineering, University of California San Diego, IEM, La Jolla, CA 92093, USA;; 16 Department of Chemical Engineering, Northeastern University, Boston, MA 02115, USA;; 17 National Institute of Standards and Technology, Center for Neutron Research, Gaithersburg, MD 20899, USA;; 18 Department of Mechanical Engineering, University of Connecticut, Storrs, CT 06269, USA;; 19 Department of Pharmaceutical Sciences, Nanotechnology Laboratory, Mercer University, Atlanta, GA 30341, USA;; 20 Knight Cancer Institute, Cancer Early Detection Advanced Research (CEDAR), Oregon Health and Science University, Portland, OR 97239, USA;; 21 Wits Advanced Drug Delivery Platform Research Unit, Department of Pharmacy and Pharmacology, School of Therapeutic Sciences, Faculty of Health Sciences, University of the Witwatersrand, Johannesburg, 7 York Road, Parktown 2193, South Africa;; 22 Faculté de Pharmacie and TIMC-IMAG (UMR 5525), University Grenoble Alpes, SyNaBi, 38000 Grenoble, France;

**Keywords:** nanotechnology, nanomedicine, biosensing, tissue engineering, microfluidics, nanotherapeutics, NanoFlorida, conference

## Abstract

The burgeoning field of nanotechnology aims to create and deploy nanoscale structures, devices, and systems with novel, size-dependent properties and functions. The nanotechnology revolution has sparked radically new technologies and strategies across all scientific disciplines, with nanotechnology now applied to virtually every area of research and development in the US and globally. NanoFlorida was founded to create a forum for scientific exchange, promote networking among nanoscientists, encourage collaborative research efforts across institutions, forge strong industry-academia partnerships in nanoscience, and showcase the contributions of students and trainees in nanotechnology fields. The 2019 NanoFlorida International Conference expanded this vision to emphasize national and international participation, with a focus on advances made in translating nanotechnology. This review highlights notable research in the areas of engineering especially in optics, photonics and plasmonics and electronics; biomedical devices, nano-biotechnology, nanotherapeutics including both experimental nanotherapies and nanovaccines; nano-diagnostics and -theranostics; nano-enabled drug discovery platforms; tissue engineering, bioprinting, and environmental nanotechnology, as well as challenges and directions for future research.

## 1. Introduction and Historical Perspective

Nanotechnology is a dynamic revolutionary force that is exponentially impacting societies around the world. Nanotechnology and nanoscience aim to create, understand, and use nanoscale structures, devices, and systems having novel properties and functions. Through ground-breaking scientific and technological advancements, nanotechnology-based products are becoming fully integrated into our daily lives. The nanotechnology revolution embodies an interdisciplinary approach and is bringing scientists together from nearly every area of scientific study. This worldwide collaboration is significantly enhancing the quality of construction materials, machinery, automobiles, electronics, renewable energy, transportation, common appliances, consumer products, entertainment, agriculture, microscopy, scientific instrumentation, health care, diagnostic assays, and medicinal drug discovery. The US government investment in the National Nanotechnology Initiative was reported as nearly $29 billion in the 2020 budget supplement [1]. The anticipated returns on investments in terms of products (>2000 consumer products) globally has been estimated at >$3.3 trillion by the year 2025, and there were over 1900 U.S.-based companies conducting R&D, manufacturing, or product sales in nanotechnology in 2016. Attracting the upcoming generation of students to this burgeoning field and training of the work force in nanoscale technologies remains a major challenge and task for the future.

For the past twelve years a consortium of Florida universities has organized an annual conference on nanotechnology to address the demands of students and faculty conducting research on different aspects of nanotechnology. This conference also serves as a forum for academic and industrial researchers to share their recent discoveries, exchange new ideas, and develop professional relationships that strengthen the state’s nanotechnology research community. The annual NanoFlorida Conference provides a venue where expert nanoscientists from across the state of Florida, the US, and the world can come together with engineers, chemists, physicists, biologists, clinicians, and students to hear about the latest research in the field and discuss new directions and collaborations. The conference provides in-depth presentations and symposia for the experienced nanoscientist, as well as background and introductory talks for students and beginning researchers interested in applying nanotechnology in their own projects and for the clinician planning trials of new drug delivery systems, diagnostic devices, and therapeutic tools. The University of South Florida organized the 12th annual NanoFlorida Conference on November 15–17, 2019. The international conference featured three keynote speakers, sixteen plenary speakers, and nearly two hundred researchers who presented their work through oral and poster presentations.

Donald Martin’s group (University Grenoble Alpes, France) provided a historical introduction to the field of nanotechnology [2]. Their discussion outlined previous concepts introduced by Zsigmondy, Schmitt, Hibbs and Feynman that provide pillars upon which we can advance our ideas about nanostructured medical devices for implantation. To put a perspective on the modern era of nanotechnology, in an introductory plenary Lisa Friedersdorf (National Nanotechnology Coordination Office, Washington DC) spoke about “Nanotechnology Now and into the Future.” She gave an overview of how the launch of the U.S. National Nanotechnology Initiative brought much excitement to the area of nanotechnology, with the promise of lightweight materials, high-density data storage, and methods to detect cancer on the cellular level. Examples of ongoing nanotechnology research, current applications, and emerging areas that will influence the future were provided. A particular focus was placed on opportunities for students and faculty to participate in and build communities of interest related to nanotechnology research and development.

## 2. Progress in Various Nanotechnology Disciplines

### 2.1. Nanotechnology Applications in Engineering

#### 2.1.1. Optics, Photonics and Plasmonics

Ryan Gelfand (Tulane University, New Orleans, LA) described details of two revolutionary new biophysics techniques based on single protein optical trapping and provided potential examples of study for furthering our knowledge of how these processes can be applied to pharmaceutical research. Elucidating protein structure, function and behavior has been instrumental in increasing our understanding of human physiology. However, many of the tools developed for determining these properties have reached their limitations. Nanoaperture optical trapping (NAOT) is a nondestructive technique used to trap a single protein without any tethering, fluorophore labeling, or adhesion to a surface for many minutes, orders of magnitudes longer than other single molecule techniques. This freedom allows the protein to move in 3 dimensions without any steric hindrance, and so we can study them close to their native environments while still trapped. When a single molecule, such as a protein, is trapped, two things change about the transmitted signal through the nanoaperture: the amplitude and the variance. This effect is due to the polarizability of the protein and a modulation of this property can be observed and studied. Rate kinetics by nanoaperture optical trapping (RK-NAOT) uses the change in signal to determine the dynamics of a trapped protein and a small molecule with nanosecond resolution. When a protein binds to a small molecule, its shape changes resulting in a change in its polarizability which is evident in the collected signal. Furthermore, by using stimulated anti-stokes Raman scattering (STARS) spectroscopy, which is a two photon method, the vibrational modes of a protein can be elucidated and its structure can be potentially determined. These are just two of the many applications that NAOT can provide to the pharmaceutical research community.

Ratka Damnjanovic (University of South Florida, Tampa, FL) presented a novel proof of concept platform for spatially and temporally precise neural excitation, using hybrid electro-plasmonic stimulation technique in a whole-cell patch-clamp configuration to elicit electrical responses in primary trigeminal neurons. Neural stimulation prosthetics utilizing electrical stimulation have limited spatial resolution due to the spread of electrical currents to surrounding tissue. To facilitate specific point stimulation, various nanomaterial-assisted neural stimulation approaches have been reported [3–14] where different localized fields are activated. Infrared stimulation has been shown to excite neurons, but with a limitation of heating surrounding tissue [15,16]. In a prior study [17], visible green light and gold-nanoparticle-coated microelectrodes have been used for plasmonic modulation of SH-SY5Y neuroblastoma cells, but with limited success and detrimental effects on cell membranes with higher levels of pure optical stimulation. To address these limitations, herein, the first cellular study of the membrane effects produced by trigeminal neurons when stimulated with plasmonic and hybrid stimulation using visible light demonstrated that various combinations of sub-threshold levels of electrical and short-duration visible light (532 nm) pulses can successfully modulate neural firing patterns. The electrical stimulus amplitude required to evoke action potentials is significantly reduced (up to 40%) when a plasmonic stimulus (1–5 V, 1–5 ms pulse) is added, compared to electrical stimulation alone. Neuron cell survival and viability after hybrid stimulation is superior to that of pure optical stimulation (72% vs. 13%). The reduction of current required to trigger action potentials, and the finding that cells stay healthy after repeated exposure to hybrid stimulation, pave the way for a novel and more versatile generation of tunable neural stimulation systems. Specifically, these capabilities can play a key role in the development of prosthetic devices including cochlear implants that offer improved frequency modulation and specificity by more selective, focused and tunable activation of auditory neurons along the cochlear frequency axis.

#### 2.1.2. Electronics

Elnaz Zeynaloo (University of Miami, Miami, FL) presented a novel mediator-free, non-enzymatic electrochemical biosensor for direct glutamate monitoring, based on the immobilization of genetically engineered periplasmic glutamate binding protein (GluBP) onto gold nanoparticle (AuNP)-modified screen-printed carbon electrodes (SPCE). Glutamate is the brain’s most abundant free amino acid and the predominant excitatory transmitter. In addition to being the influencer of a variety of behaviors such as sensory perception, mood, and motor control, an excess of glutamate can cause excitotoxicity [18], which is a common pathological process in many neurologic disorders such as stroke [19], brain trauma [20], and Alzheimer’s disease [21]. Among various glutamate detection and monitoring technologies, electrochemical methods have shown great potential in point-of-care applications due to their high performance, easy miniaturization, low cost, and small sample volumes [22,23]. Enzymatic electrochemical biosensors are mostly used in glutamate detection [24–26]. However, enzyme-based electrochemical sensors still suffer from limitations, such as an indirect quantification of analytes, a requirement of redox mediators, and strong matrix interference (ascorbic acid) [27]. Cyclic voltammetry was performed to determine the glutamate and common interfering substrate concentration in a 50 mM sodium phosphate buffer solution (pH = 7.4). The results showed an excellent sensitivity with a detection limit of 0.1 μM and a linear range of 0.1 μM–1 μM of glutamate concentration. The sensor specificity was tested with a series of interfering substances, including amino acids (aspartate, glutamine, serine, and lysine) and a common matrix interference (ascorbic acid). The biosensor exhibited high selectivity toward glutamate over those substances, thus demonstrating its potential applications in biomedical and pharmaceutical analysis.

### 2.2. Biomedical Devices

Ratnesh Lal (UCSD, San Diego, CA) discussed wireless electronic nano-biosensors for global health and security. Early and rapid diagnosis of biomarkers for diseases and transmissible infectious pathogens, including viruses, toxins, and bacteria inflicting large scale abnormalities and fatalities as well as plant diseases, are essential for effective global health and security. Existing approaches for the large scale assay of biomarkers use optical sensing, require amplification of the bio-sample, are time and resource consuming, require large laboratory setup, are non-portable, and lack sensitivity. On the other hand, nanodevices integrating nano-bio-probes and advanced nano-electronics with wireless capability as an array-biosensor platform on chips, with single molecule sensitivity and specificity, would overcome the abovementioned limitations of the current approaches. They can identify and monitor simultaneously an array of biomarkers of genetic defects, viruses, bacteria, toxins, and infectious airborne pathogens. The detected signals can be transmitted by Bluetooth to personal electronics, including smart phones, tablets, and computers. Lal presented an example of an integrated platform combining DNA nano-tweezers (a dynamic DNA nano-device) as a nucleic acid-sensing probe, electrical biosensors (using graphene FET), and an analytical wireless communication system [28]. The electrical signal resulting from the resistance changes triggered by the interaction between DNA nano-tweezer probes and defective DNA samples was recorded and transmitted remotely. Significantly, this new approach is label-free and does not require amplification of the probes to improve the detection sensitivity or an expensive immovable lab setup [29,30]. As such, implementation of this technology would allow cheaper, faster, implantable or wearable or hand-held, and portable point-of-care health status monitoring and accurate diagnoses of cancer, degenerative, genetic, and various communicable diseases as well as early detection of pathogens with potential for large population casualty.

**Figure 1.**
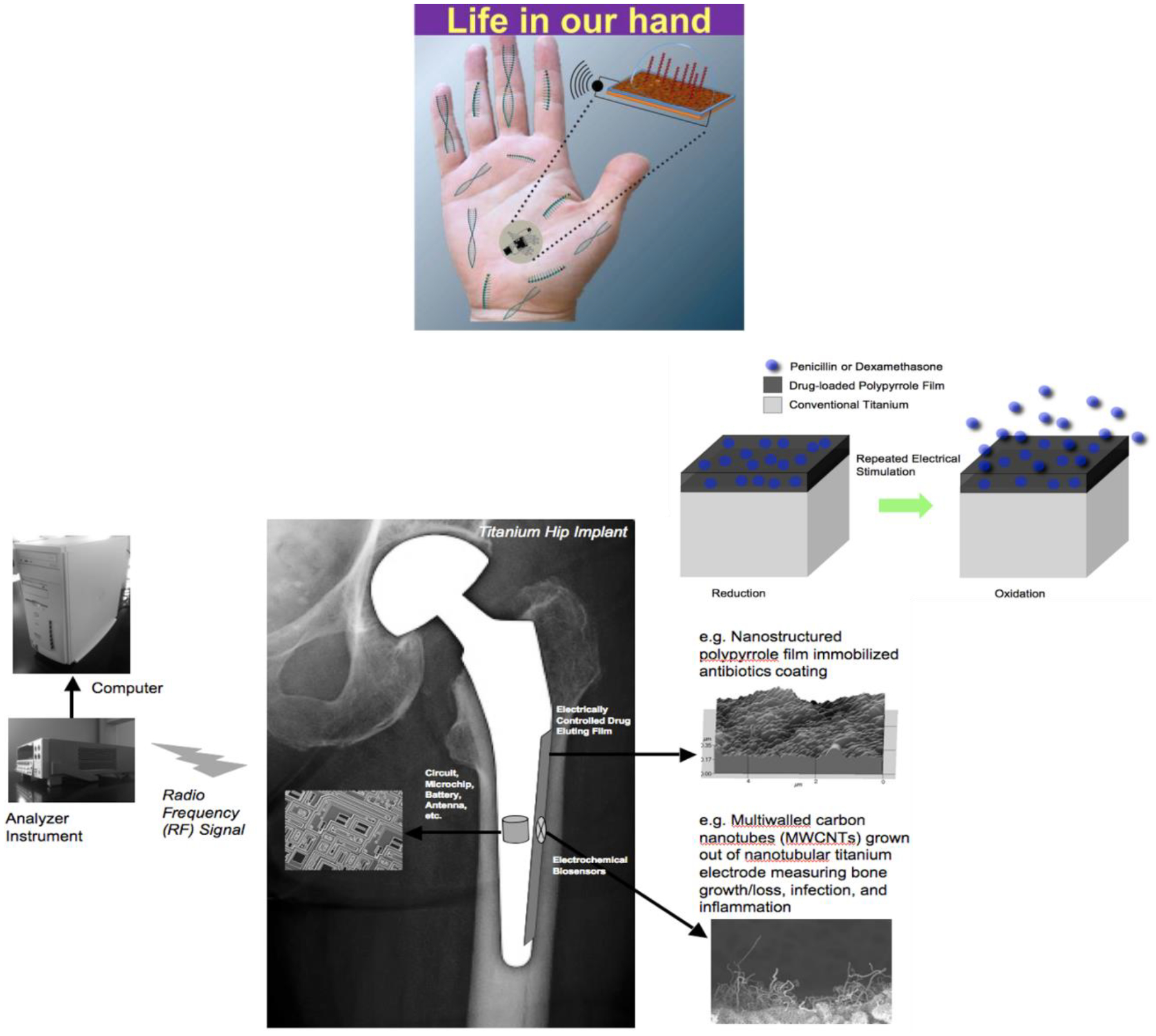
The SMARTHIP^™^ can detect cell presence and function next to a hip implant in real time and possesses on-demand release of nanoparticles, antibiotics, anti-inflammatory agents, and more to ensure implant success. Further, all information can be sent to a hand-held device to provide personalized medicine.

Thomas Webster (Northeastern University, Boston, MA) discussed how nanotechnology can be used to increase tissue growth and decrease implant infection without using antibiotics but using sensors. His group has shown that nanofeatures, nano-modifications, nanoparticles, and most importantly nanosensors can reduce bacterial growth without using antibiotics. He summarized techniques and efforts to create nanosensors for a wide range of medical and tissue engineering applications, particularly those that have received FDA approval and are currently being implanted in humans [31]. Currently, there are a dozen or more ways to incorporate nanostructured features onto implants, changing many fundamental properties including surface area, surface chemistry, hydrophilicity, radio opacity, roughness, and catalytic properties that in turn affect surface energy and mechanical and electrical (conductivity) properties of the implanted materials such as catheters, stitches, or implants that are inserted for wound healing devices for the skin [32]. Interestingly, his lab developed a quantitative equation that can predict material protein and material cell behavior, so that one does not have to do this by trial and error [33]. Also, he discussed the use of micro- and nano- shot peening of stainless steel toward incorporating nanoscale surface features and nanoscale grain sizes which in turn increase osteoblast function and decrease bacterial growth, not killing mammalian cells but inhibiting bacteria cells, without drugs. Finally, he described the smart sensors in orthopedics that his group has been studying, which have three components: a component that senses what is going on in the body, a processor that processes the information and sends it to a computer or an iPhone, and a user interface where that sensor can then respond to the problem such as by increasing bone growth, decreasing infection, or decreasing scar tissue. He provided the example of a hip implant comprised of carbon nanotubes. These carbon nanotubes can measure the conductivity of the cell that attaches to the implant, and thus can determine whether it is a bone cell or a bacterial or an inflammatory cell. The hip implant also incorporates a wireless technology so that the sensor can then communicate the current status to a computer to inform the patient and the surgeon. Finally, a responding unit is incorporated made up of a polymer that can degrade under an applied electromagnetic field to release nanoparticles that could kill the bacteria, release bone growth factors that could increase bone growth, or release an anti-inflammatory agent if inflammation is the problem (Figure 1).

**Figure 2.**
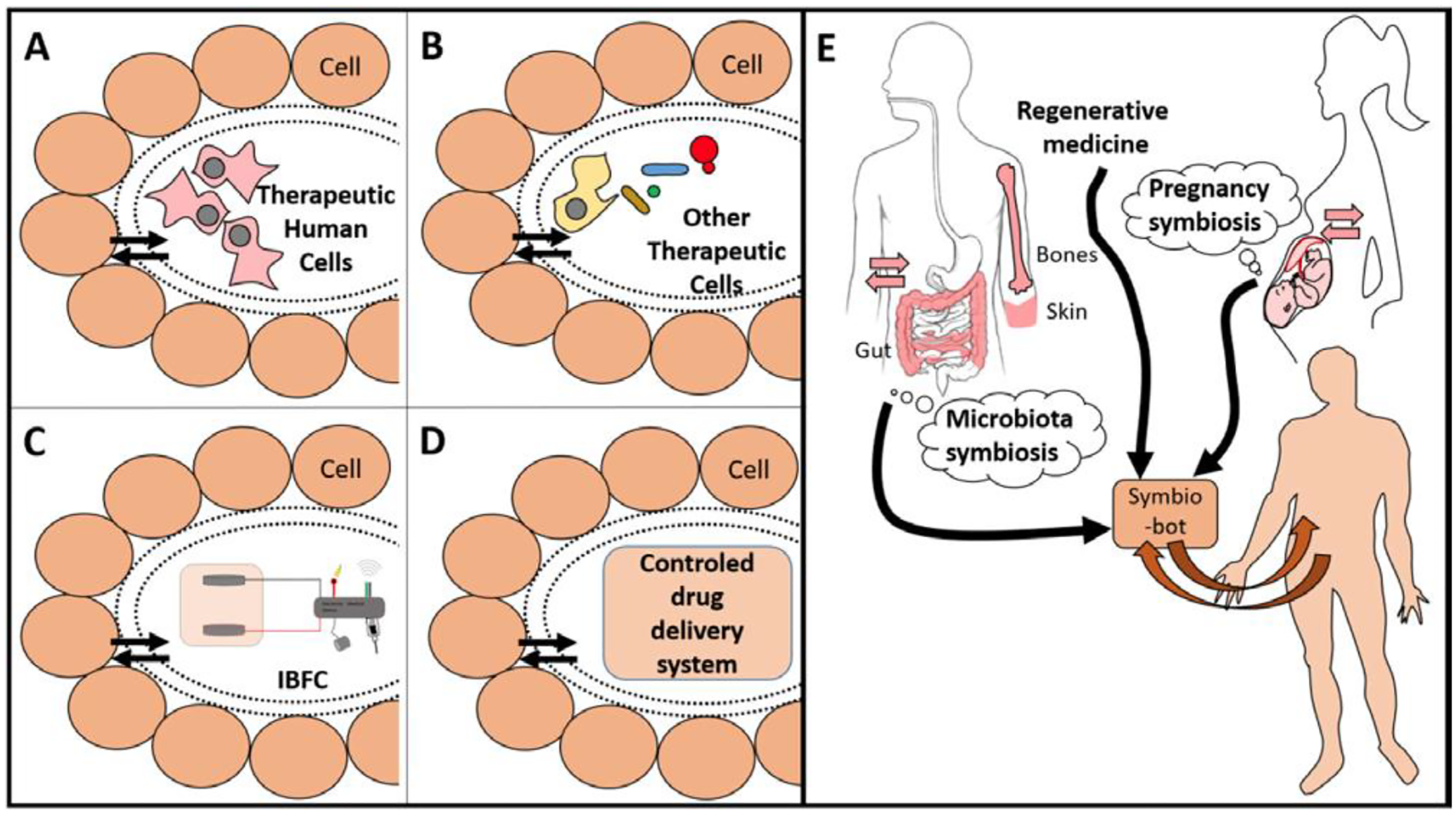
Examples of symbio-bots **(A-D**) that can be created in a bioinspired way (**E**). Each device is separated with a smart porous packaging that allows for duplex communication. Therapeutic cells (**A,B**) need a porous encapsulation that avoids an immune reaction and allows protection from both sides. They may be human cells, as MSC or specialized cells such as β-cell from Langherans islets (**A**), or other eukaryotic or prokaryotic cells (**B**). Panel (**C**) shows an IBFC linked to an electronic medical device. Panel (**D**) shows a generic device delivering a therapeutic molecule. Panel (**E**): Existing symbioses (i.e., microbiota or pregnancy) are a source of bio-inspiration to establish a duplex communication between the body and its implants. Regenerative medicine should embrace this concept of bioinspiration for better design and integration of implants, especially for future symbio-bots. (Reproduced with permission from [34].).

Donald Martin (University Grenoble Alpes, France) described the concept of “symbiotic nanostructured medical devices,” taking into account the fact that the implanted device is intended to have some communication of energy or materials with the body. If this is one-way communication from the device to the body, such as for a drug delivery, the challenge is to avoid the degradation or encapsulation of the device. However, if the implanted device is intended to restore body or organ function, then it needs to mimic the two-way (duplex) communication that is required for transplanted living organs or cells [34]. Examples of these implantable duplex communicating systems include biofuel cells or open-loop feedback devices where a molecule from the body is utilized by the duplex communicating system to produce a different material (e.g., molecule or energy) for use by the body. The challenges for such symbiotic systems are to be biocompatible and to maintain two-way transport communication of materials. Symbiotic devices extend the classical biocompatibility concept to include this functional requirement for continuous two-way (duplex) communication of materials with the body. It is likely that by utilizing such a biomimetic approach, scientists can be more efficient in mimicking what is happening at a molecular and cellular level to create a porous membrane that allows long-term exchanges of molecules between an implanted device and the body (Figure 2). Having this novel concept in mind will guide the research in a new field between medical implant and regenerative medicine to create actual symbiotic devices.

### 2.3. Nano-Biotechnology

David Hoogerheide (National Institute for Standards and Technology [NIST], Washington DC) described the use of neutron scattering for nano- and biomaterials. Neutron scattering is a powerful, versatile tool for both solid state and soft material characterization. Neutrons are a particularly good probe for the light elements that compose biological materials, enabling detailed structural characterization over length scales from 1 to 1000 nm. Because neutrons interact with nuclei rather than the electronic structure of materials, individual material components can be distinguished using isotopic substitution, which is often as simple as exchanging heavy for light water. The suite of neutron scattering techniques includes probes of high- and low-resolution structures as well as dynamics on the pico- and nano-second time scales (see Table 1) and are available domestically at the user facilities at the NIST Center for Neutron Research and Oak Ridge National Laboratories High Flux Isotope Reactor and Spallation Neutron Source.

**Figure 3.**
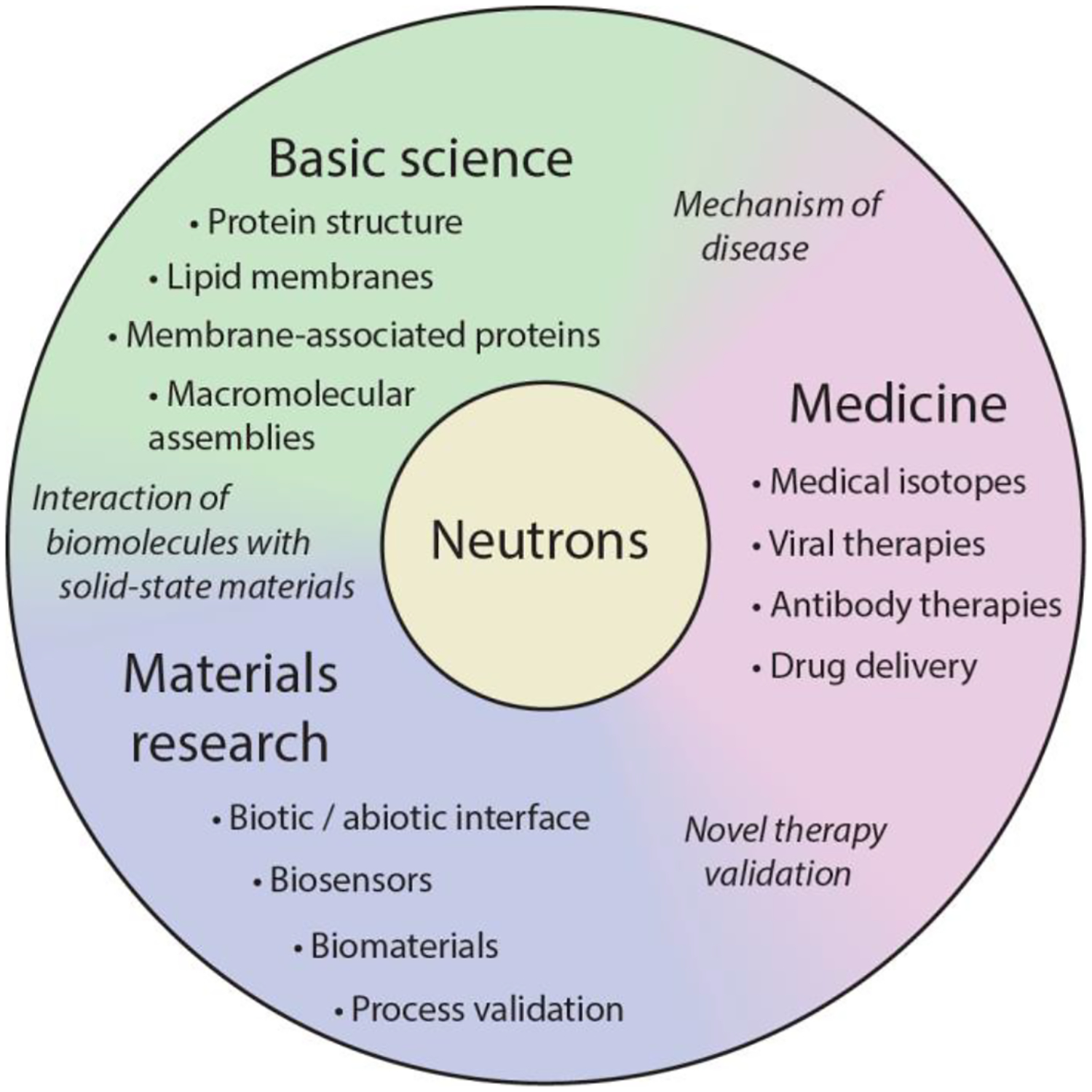
Application space of scattering neutrons from biological materials. Applications at the intersections of basic science, materials research, and medicine are italicized.

The application space for neutron scattering techniques is shown schematically in Figure 3 and comprises basic science (“structural biology”), materials research, and pharmaceutical medicine. In structural biology, neutron scattering, often in combination with molecular perdeuteration, is widely used to determine the structural arrangement of macromolecular complexes of lipids, proteins, and nucleic acids. In a recent example, the molecular surface of dimeric tubulin responsible for adsorption to a supported lipid bilayer was determined using neutron reflectometry [35]. As an example of neutron scattering from biosensor materials, neutron reflectometry was used to characterize the thin film structure of a molecular layer deposition film used in construction of next-generation DNA sequencing devices [36].

### 2.4. Nanotherapeutics

#### 2.4.1. Experimental Nanotherapies

Eleni Markoutsa and associates (University of South Florida, Tampa, FL) presented their work on the development of a shell-core multifunctional nanosystem with a dual payload: a plasmid construct encoding for shRNAs against NS1 and P, and an anti-fusion peptide (HR2D) and anti-ICAM antibody as a targeting moiety. Their studies in vitro showed the effectiveness of the NS1/P shRNAs and HR2D peptide to successfully inhibit respiratory syncytial virus (RSV) infection. Biodistribution studies revealed high accumulation and prolonged retention of the nanosystem in the lung and targeted to RSV infected cells. Studies in an immunocompromised mouse model showed high efficacy of this system to prophylactically inhibit viral fusion and replication, and effectively treated an established infection. Furthermore, this nanosystem modulated the expression of Th2 and allergy-associated cytokines such as IL4, IL-13 and IL-17 indicating a direct role of this nanosystem in the mechanisms involved in the regulation of disease pathogenesis. These results demonstrate the potential of this nanosystem as both a prophylactic and therapeutic treatment against RSV, which could profoundly impact the health of high risk infants as well as vulnerable elderly populations.

Rinku Dutta (University of South Florida, Tampa, FL) presented results on novel nanoparticle (NP) formulations for colon-targeted delivery of chemotherapeutics. Most current therapies suffer from dose-limiting toxicities, low bioavailability, poor drug loading, and uncontrolled non-specific delivery [37]. Oral drug delivery is the preferred route due to patient self-administration compatibility, ease of manufacture, and cost effectiveness [38]. Hydrophobic drugs can be formulated in NPs for better drug dissolution and absorption through the oral route. Dutta’s lab team has previously shown the anti-cancer properties of the antibiotic Mithramycin (Mit-A) in vitro and in vivo [39]. However, the drug is highly lipophilic, making it difficult to formulate for effective delivery [40]. Using the marker CD133, a cancer stem cell marker that is overexpressed in colorectal cancer [41], they have developed antibody-conjugated Mit-A loaded nanoparticles (NPs) for colon targeted oral delivery and the treatment of colorectal cancer. The NPs were embedded in a chitosan/alginate hydrogel matrix and orally gavaged to athymic nude mice. A strong NIRF signal was observed in the ascending colon along with the cecum and slightly in the descending portion post 6 h, and it decreased with time with no signal detected at 24 h due to rapid clearance. Thus, the DiR-loaded particles have shown time dependent delivery of a payload at the cecum of athymic nude mice post oral delivery. The antibody-modified drug loaded nanoparticles hold dual potential for targeting and therapeutic benefits in the treatment of colorectal cancer.

#### 2.4.2. NanoVaccines and Therapies

Thanh Duc Nguyen (University of Connecticut, Storrs, CT) presented his team’s research creating 3D microstructures of biodegradable polymers for developing single-administered vaccines and converting the biopolymers into “smart” piezoelectric nanomaterials, which can generate electricity under deformation and vice versa, offering a variety of exciting applications in biodegradable force sensors, tissue-engineering scaffolds, and medical transducers [42–45]. Bioresorbable and biocompatible smart polymers can be used as medical suture materials, biosensors, and tissue engineering scaffold by changing their form, shape, or structure at the micro- and nanoscale level. Common vaccines, such as polio vaccine and hepatitis B vaccine, require three injections, while insulin for diabetes and growth hormone therapy require injections on a daily basis. Multiple injections of drugs have many disadvantages; they are painful and inconvenient for patients, produce a large amount of biohazard waste, need special transportation and storage facilities, and often cause recurrence of infections. Polymers like polylactic acid (PLA) and poly(lactic-co-glycolic acid) (PLGA) can be used for developing a single injection multi pulse drug delivery system where a drug is loaded inside the resorbable PLGA shell in an injection and these core shell micro particles will be released at different time points in a multi burst release fashion. This delivery method still requires injection, and another problem is the presence of large particles in the injection which can be surmounted by the SEAL (StampEd assembly of polymer layers) technique. SEAL is a filling free, high throughput, automatable fabrication method to produce microneedles for the injection of free and single administration of vaccines. Here drugs are aligned and loaded into core shell biodegradable microneedles depending on molding and computer chip alignment-based processes. This kind of microneedle has been developed for a pneumonia vaccine, which has shown better immune response and percent survival rate compared to the commercially available vaccine PREVNAR. Currently existing pressure sensors require invasive removal of the implanted device because of their nondegradable nature. However, in recent work biodegradable PLGA has been converted into piezoelectric nanofibers which can be used as a biosensor for detecting pressure in different areas (intra-cranium, cartilage, intra-abdomen, etc.) of the body.

Martin D’Souza (Mercer University, Atlanta, GA) discussed his work on the design and delivery of nano- and micro-particulate vaccines, delivered transdermally via microneedles for both infectious diseases and cancers. This innovative, inexpensive, and painless method of vaccine delivery technology is rather broad based and can be used to administer multiple vaccines in a single vaccine patch applied to the skin much like a “band-aid-patch” [46]. On the infectious disease vaccine front, they have been working on nano- and micro-particulate vaccines for several infectious diseases such as (a) universal influenza, (b) HPV, (c) RSV, and (d) gonorrhea vaccines designed for delivery via microneedles [47]. The vaccine antigen was formulated into microparticles with the aid of a spray dryer, using bio-degradable and bio-compatible matrices [48]. These vaccine particles were then incorporated into micro-needles for delivery via the transdermal route. Animals were challenged with live virus/bacteria to determine the level of protective immunity developed after vaccination. Further, the team examined the expression of co-stimulatory molecules that impact antigen presentation in human macrophages pulsed with the antigen. The up-regulation of other co-stimulatory molecules such as CD-40 and CD-80 was also determined. In conclusion, the novel particulate vaccines are robustly taken up by antigen presenting cells and up-regulate co-stimulatory molecules that enhance antigen presentation, which is a pre-requisite for inducing adaptive immunity. The other innovative microneedle particulate vaccines under study include therapeutic cancer vaccines such as (a) breast, (b) melanoma, (c) ovarian, and (d) prostate cancer vaccines.

#### 2.4.3. Drug Discovery Platforms

Motivated by a desire to understand biological compartmentalization and to use it for biotechnology, Steven Lenhert (Florida State University, Tallahassee, FL) described the fabrication and application of arrays of micrometer and nanometer scale lipid droplet microarrays. If micro-wells were the size of biological vesicles, there would be room for 10^17^ compartments in one cubic centimeter. In contrast, the state of the art in high-throughput screening uses 1536 well plates and about 3 mL of solution per plate. Arrays are fabricated by nanointaglio, which involves the transfer of fluid inks from the recesses of a microstructured stamp onto a substrate [49]. Thousands of different materials can be integrated into the droplets using pin spotting technology. Lipophilic small molecules can be encapsulated into the oil droplet, and cells cultured over the arrays for phenotypic screening with pharmaceutical applications [50]. Furthermore, exposure of these droplet arrays to lipid binding analytes such as proteins while observing scattered light from the arrays allows label free detection of remodeling events in the lipid droplet nanostructure [51]. Droplet size, shape, and composition are central to these applications. Recent progress in the use of these multiphase fluids to investigate and control biological systems was presented.

### 2.5. Nanodiagnostics and Nanotheranostics

Mustafa Çulha (Oregon Health & Science University, Portland, OR) described his team’s effort to utilize surface-enhanced Raman spectroscopy (SERS) for label-free cancer diagnosis from “liquid-biopsy” at The Knight Cancer Institute’s Cancer Early Detection Advanced Research Center, or CEDAR, in addition to the use of the technique for tissue differentiation [52–54]. SERS, a very sensitive mode of Raman Spectroscopy (RS), can provide label-free fingerprint molecular information from a molecular mixture. Thus, in theory, RS can be used for the differentiation of healthy and diseased tissues and body fluids. RS and almost all of its modes have long been investigated for diagnosis, and their potential is well-demonstrated in the literature [55,56]. In contrast to RS, a gold or silver nanostructured noble metal surface is used to enhance Raman scattering up to 10^11^ in SERS [57]. Since its discovery during early 1970s [58,59], it has been applied for the detection of numerous analytes with biological and nonbiological origins. In recent years, it has also been investigated for its potential in label-free cancer diagnosis. In “liquid biopsy” studies, Çulha’s team utilized mesoporous silica coated silver nanoparticle (AgNPs@Si) to exclude molecular structures and allow small metabolites to filter onto the AgNPs surface for SERS detection. The results of these studies suggest that the approach can be used to detect cancer from a “liquid biopsy.” However, the detection system needs to be validated through clinical trials and the study continues in this direction.

### 2.6. Tissue Engineering

Tao Wang (University of South Florida, Tampa, FL) presented his team’s efforts to build a microfluidic sensor platform for 4D tumor cell culture monitoring. The aim was to recapitulate the complex 3D structure, heterogeneous cell environment, and cell-cell interactions found in vivo by combining in vitro microfluidics technology, sensor technology, and 3D cell culture techniques. This platform advances current cell culture techniques and offers an improved model to study the tumor microenvironment. There is growing awareness that static 3D tumor-organoids, or “tumoroids,” have different biochemical properties and gene expression profiles compared to in vivo conditions [60]. Static culture techniques lack the nutrition exchanges and shear stress present in the dynamic in vivo environment [61,62]. To overcome these challenges, an optimized surface acoustic wave (SAW)-based multiple-layer sensor and passive electrode potential sensor was integrated with microfluidic 96-well plates and fabricated by traditional microfabrication techniques. Full optimization of the sensor settings and the flow rate was conducted by finite element analysis to increase the sensitivity and generate appropriate shear stress on cells [63–66]. It was found that a fiber inspired smart scaffold (FiSS) platform established in the Mohapatra laboratory allows for the growth of 3D tumor-like aggregates (tumoroids), which resemble in vivo tumors [67]. The FiSS culture environment creates a more representative model of tumor growth and drug resistance compared to 2D cultures that lack the ability to mimic the tumor microenvironment. These tumoroids also allow for the expansion and study of cancer stem cells, which are a small population of cancer cells within the tumor that play critical roles in the development of drug resistance, metastasis, and cancer [68]. The FiSS was placed into the microfluidic 96-well plate and used to culture HT-29, HCT116 (human colon cancer), and Lewis Lung carcinoma cells. The static culture was compared to perfused culture via standard molecular biology techniques, while cell metabolism was assayed by the integrated SAW and pH sensors. This 4D platform for tumoroids showed higher drug resistance, enhanced cancer stem cell properties, and increased secretion of pro-tumor cytokines compared to a monolayer or static 3D culture model. The perfused microenvironment represents an improved culture model for drug compound efficacy/toxicity screening for personalized cancer treatment.

### 2.7. Bioprinting

Pierre Kondiah (University of the Witwatersrand, Johannesburg, South Africa) presented the significance of personalized 3D printed oral tablets for overcoming the limitations of conventional tablet fabrication approaches, as well as specifically tailoring drug delivery particularly where a patient response to the same drugs is varied [69–72]. 3D printing is rapidly forging its niche as an advanced and transformative build technology holding significant application in pharmaceutical sciences for creating bioinspired solid 3D devices from a digital model with customizable, complex shapes, surfaces, and architectures employing diverse materials. Over the past 15 years, 3D printing expanded into the healthcare industry, affording convenient, cost-effective manufacturing of personalized pharmaceutical, medical, and dental products as a pertinent advantage over traditional manufacturing methods. The noteworthy applications of 3D printing in pharmaceutical research include the design of customizable 3D-printed oral tablets, drug delivery devices, and tissue engineering scaffolds. These applications are a focus of the research undertaken at the Wits Advanced Drug Delivery Platform (WADDP) research unit. Working with Professor Pillay and Professor Choonara, they have designed conceptual models, customizing the mechanism by which the research team achieves this, through the use of each patient’s individual information to produce their optimal drug dose and release rate. Recently, drug delivery system design, as demonstrated through projects in the Research Unit, has been largely fueled by polymer science resulting in extended- and delayed-release tablets, transdermal systems, and long-acting implants. His team employed 3D printing to introduce a novel element into dosage form development (i.e., digital control over the arrangement of matter) to create unique delivery systems with striking alterations in immediate-release, modified-release, and combination-drug delivery systems. This facilitates targeted, controlled, and customized drug release, which can be achieved, for example, by printing a complex construct of polymeric stimulus-responsive or barrier layers or using computer software to generate a template that achieves the desired release in the specific patient population to enhance treatment efficacy and patient compliance. The first FDA-approved 3D printed tablet for epilepsy, Spritam, is now available in the US. This type of technology offers more accessibility for the future in relation to drug delivery devices and implants.

Bioplotting is a specialized arm of 3D printing that employs biological materials in computer-aided tissue engineering. This creates structures for enhanced biomimicry. The goal of this therapy structure is to achieve an effective combination of cell and bioactive components for restoration and/or improvement. When developing 3D printed tablets, there is a fixed drug dose that contains a combination of anti-HIV drugs. Within the combination, there are three specific drugs that are included in a fixed-dose tablet creating a non-enhancement. This provides a conventional bioink that the drug is promoting. Research shows that within 10–12 h, there is a peak concentration within these drugs that causes sweating of polymers. The addition of anti-inflammatory agents to the 3D bioink scaffolds provides acute treatment of specific inflammation and support for adequate tissue repair and rehabilitation. The specific prepared scaffold, PCL-PVA-PAA (PPP), experienced load bearing tissue repair where inflammation was prevalent [70].

Studies are also under way on a wound healing device with specific wound regenerating properties incorporating cell seeding and growth factors. This device would provide controlled released active compounds to the tissue to aid in regeneration. A 3D print was obtained using a UV light. Another device currently being researched is the use of a pseudo-bone drug delivery system to be implanted at the site of the bone fracture. The system would be comprised of copolymeric biometric and a thermogel. The thermogels were designed to be loaded with a statin drug and then optimized for 3D bioprinting by using MATLAB. Studies continue to test the response to the duration of the release of simvastatin and thermo-gelation temperatures [69–72].

### 2.8. Environmental Nanotechnology

Dong Xiang (University of Florida, Gainesville, FL) presented research with an overall goal to develop a sensor-analytics point solution (SNAPS) for monitoring soil nitrogen status (NH_4_^+^ and NO_3_^−^ ions) to be used for providing on-farm decision support [73]. Soil is a living resource that is vital to global function, and acts as an interface between agriculture and the environment. The key indicator in soil, nitrogen, is spatially and temporally dynamic, varying widely with soil type and local conditions. Nitrogen is required to sustain agricultural productivity and maintain ecological function. While many tools have been developed for tracking soil nitrogen status, there are few commercial sensors with specificity and durability for long-term monitoring and decision support. Laser-scribed-graphene (LSG) electrodes were first fabricated using a biomimetic entropic patterning technique recently developed by the McLamore lab at UF, and then the electrodes were functionalized with ion-selective polymer composites [74]. Various hardware configurations were developed for providing wireless functionality in scenarios of short-term and long-term monitoring [75], and sensors were validated in laboratory soil column studies based on signal acquisition with a smartphone. A smartphone-based analytical tool was developed for determining whether water meets runoff guidelines using block coding (Thunkable). The sensors demonstrated near Nernstian sensitivities (51 to 54 mV/decade) with a detection limit in the range of 20 to 25 uM, and low drift (1 to 5 μV/hr) [74]. A proof-of-concept SNAPS tool is currently being tested in field studies and the results of these trials will be used to improve the hardware/software configuration for the tool prior to large scale field testing. SNAPS have the potential to help resource managers or growers better manage fertilizer applications by providing on-farm decision support.

Victoria Morgan (University of Florida, Gainesville, FL) presented a low-cost and quick-analysis framework by coupling inexpensive nanosensors for inorganic mercury determination with a risk model on a mobile device to equip communities with real-time information for decision support. Mercury is a dangerous neurotoxin that can cause a plethora of health effects at levels as low as two parts per billion for inorganic forms in drinking water [76]. These effects are often heightened in low-income artisanal small-scale gold mining communities where there is less control over environmental and occupational exposure and the resources to monitor mercury pollution [77]. Standard laboratory techniques to test the water quality (e.g., atomic absorption spectroscopy) are accurate but expensive and impractical [78]. In addition to inconvenient sensing technologies for resource-limited settings, communities often lack post hoc analysis tools for characterizing the risk of contaminants [73,79]. Electrodes were fabricated by laser scribing polyimide and decorated with nanometals, such as nanocuprous oxide, to create carbon-metal nanohybrid structures. The electrochemical behavior and sensor performance were analyzed via differential pulse voltammetry on a portable ABE-STAT potentiostat [75]. Distinct peaks of current as a result of the addition of mercury concentrations were linear from 0–1000 ppb with a response time of 1 min. The sensors were paired with a hazard quotient calculator application on a smart device to create a participatory monitoring effort for making informed decisions in the region. This information is being used to develop mitigation strategies and protect groups vulnerable to mercury toxicity. The methods for developing the carbon nanosensors and decision support tools do not require specialty equipment and are facile, economic, and quick, which makes this method useful for development in rural areas. Sensors, risk models, and decision support analyses present a new paradigm shift in participatory monitoring for disadvantaged rural communities.

## 3. Challenges and Future Directions

While many of the conference presentations highlight future directions in various fields of nanotechnology research, there remain several challenges. Despite the rapid development and escalating publications in this field, there remain a number of challenges in product development and commercialization.

One of the key issues is the health, safety and environmental aspects of some nanomaterials and their regulatory status. The responsible utilization and adoption of new technology will be impacted either favorably or unfavorably by the regulatory requirements for its oversight and further development. These regulatory requirements can either provide a platform which facilitates decision-making and responsible technology adoption or create unnecessary barriers to innovation and technology utilization. Regulations are aimed at identifying potential risks while avoiding unnecessary data generation, time delays, and increased costs. To this end, over the last two decades US agencies such as the Environmental Protection Agency (EPA) and the Food and Drug Administration (FDA) have conducted evaluations of nanomaterials with respect to environmental sustainability across the life cycle and the challenges that remain in developing and applying scientific information to determine the safety of manufactured nanomaterials. In addition, international governance bodies are evolving to address the benefits of nanotechnologies while seeking to manage their potential risks to human health and the environment through a variety of voluntary, standard-setting, regulatory, statutory, and related governance platforms [80].

Particularly in relation to nanotherapeutics, a major issue has been that the Rule of Five that has been successfully used to predict the safety/toxicity of low molecular weight therapeutics developed for oral administration does not apply to nanomaterials. Indeed, the majority of nanomaterials in existence violate at least two or three of these rules [81]. Thus, manufacturers need to be vigilant and careful in the design and deployment of this useful class of novel materials.

Finally, there is a need to develop an integrated approach in order to balance appropriate regulatory oversight versus technology adoption, which was successfully used by DuPont [82] and comprised the development of a nano risk framework for responsible development involving a shared responsibility among all stakeholders including researchers, manufacturers, and regulating bodies. Then only can translational nanotechnology live up to its estimated return on investment.

## Figures and Tables

**Table 1.** Suite of neutron scattering techniques for probing biological materials.

Technique	Sample Form	Length (Time) Scale	Information
Small angle neutron scattering (SANS)	Solubilized	1 nm–10,000 nm	Structure
Neutron reflectometry (NR)	Thin film	1 nm–1,000 nm	Structure
Neutron macromolecular crystallography (NMX)	Crystallized	0.1 nm resolution	Atomic structure
Neutron spin echo (NSE)	Solubilized	0.1 nm–100 nm (0.01 ns–1000 ns)	Collective dynamics
Inelastic neutron scattering	Powder	0.5 nm–5 nm (0.01 ns–5 ns)	Diffusive dynamics
